# Prenatal and Early Postnatal Exposure to Cigarette Smoke Decreases BDNF/TrkB Signaling and Increases Abnormal Behaviors Later in Life

**DOI:** 10.1093/ijnp/pyv117

**Published:** 2015-10-26

**Authors:** Lan Xiao, Vincent L. Kish, Katherine M. Benders, Zhong-Xin Wu

**Affiliations:** Department of Neurobiology and Anatomy, Robert C. Byrd Health Sciences Center, West Virginia University, Morgantown, WV

**Keywords:** Brain-derived neurotrophic factor, tropomyosin receptor kinase B, pregnancy smoking.

## Abstract

**Background:**

Cigarette smoke exposure during prenatal and early postnatal periods increases the incidence of a variety of abnormal behaviors later in life. The purpose of this study was to identify the possible critical period of susceptibility to cigarette smoke exposure and evaluate the possibe effects of cigarette smoke during early life on brain-derived neurotrophic factor/neurotrophic tyrosine kinase receptor B signaling in the brain.

**Methods:**

Three different age of imprinting control region mice were exposed to cigarette smoke or filtered air for 10 consecutive days beginning on either gestational day 7 by maternal exposure, or postnatal days 2 or 21 by direct inhalation. A series of behavioral profiles and neurotrophins in brain were measured 24 hours after mice received acute restraint stress for 1 hour on postnatal day 59.

**Results:**

Cigarette smoke exposure in gestational day 7 and postnatal day 2 produced depression-like behaviors as evidenced by significantly increased immobility in both tail suspension and forced-swim test. Increased entry latencies, but not ambulation in the open field test, were also observed in the gestational day 7 and postnatal day 2 cigarette smoke exposure groups. Genetic analysis showed that gestational day 7 cigarette smoke exposure significantly altered mRNA level of brain-derived neurotrophic factor/tyrosine kinase receptor B in the hippocampus. However, behavioral profiles and brain-derived neurotrophic factor/tyrosine kinase receptor B signaling were not significantly changed in PND21 cigarette smoke exposure group compared with FA group.

**Conclusions:**

These results suggest that a critical period of susceptibility to cigarette smoke exposure exists in the prenatal and early postnatal period, which results a downregulation in brain-derived neurotrophic factor/tyrosine kinase receptor B signaling in the hippocampus and enhances depression-like behaviors later in life.

## Introduction

Cigarette smoking is common among pregnant women (Allen et al., 2008; T. Liu et al., 2011; Cornelius et al., 2012; Elmasry et al., 2014). Ten to fifteen percent of pregnant women continue smoking despite multiple adverse outcomes in offspring (Mendola et al., 2002; Cornelius et al., 2011; Amos-Kroohs et al., 2013). Maternal smoking during pregnancy has been reported associated with abnormal behaviors and cognitive development in offspring, and these adverse effects on neurodevelopment might persist through adolescent period and extend into adulthood (Weitzman et al., 1992; Barros et al., 2011; Cornelius et al., 2011; T. Liu et al., 2011; Cornelius et al., 2012; Elmasry et al., 2014). The probability of developing psychiatric disorders increases in children whose mothers smoke cigarettes during pregnancy, suggesting a possible critical period of developmental sensitivity to cigarette smoke (CS) exposure exists during the prenatal and early postnatal period.

The central nervous system develops rapidly during fetal and early postnatal life. Given the dynamic and vulnerable nature of developmental processes, this period of morphogenesis is likely to be exquisitely sensitive to environmental insults (Slotkin et al., 2002; Dwyer et al., 2009; La Maestra et al., 2011; Amos-Kroohs et al., 2013; Balsevich et al., 2014). Although little is known about how prenatal and early postnatal smoking exposure influences brain development, emerging studies have shown that neurotransmitters are changed in nicotine and cigarette smoking animals (Slotkin et al., 2002, 2006; Parameshwaran et al., 2012). In animal models, decreased brain weight, cortical thickness, and neural density were found in prenatal CS-exposed groups (Roy and Sabherwal, 1994). Similarly, clinical studies have also shown that prenatal CS exposure resulted in a reduction in fetal head growth and cerebellar development (Roza et al., 2007; Rivkin et al., 2008; Ekblad et al., 2010; El Marroun et al., 2014). The imaging studies further found the decreased frontal lobe, frontal cortex, and parahippocampal cortices in neonates with CS exposure (Ekblad et al., 2010; Haghighi et al., 2013; J. Liu et al., 2013). In addition, reduced cerebral cortical gray matter and cortical gray matter, including amygdala and thalamus in CS-exposed children, were also found (Rivkin et al., 2008). Although these studies have demonstrated prenatal CS exposure correlates with abnormal brain function and morphology, the signaling cascade underlying these alterations still remains unclear.

Neurotrophins, a group of polypeptide growth factors, includes nerve growth factor, brain-derived neurotrophic factor (BDNF), neurotrophin-3, and neurotrophin-4 (Huang and Reichardt, 2001). BDNF is the most important neurotrophic factor that promotes and maintains growth and survival of the central nervous system (McAllister, 2001; Soule et al., 2006; Cowansage et al., 2010; Gomez-Palacio-Schjetnan and Escobar, 2013). The function of BDNF is mainly mediated by binding to its receptors, such as tropomycin receptor kinase (TrkB) family of tyrosine kinase receptors (Lu et al., 2005; B. H. Lee and Kim, 2010). Disruption of normal synthesis and release of BDNF has been reported to associate with a variety of behavioral abnormalities (Nibuya et al., 1995; Tang et al., 2008; Tuon et al., 2010; Yochum et al., 2014). Compromised function of plasticity and BDNF signaling has been implied in the pathophysiology of depression. Duman and coworkers (1997) have shown that synthesis of BDNF was increased by antidepressant treatment, suggesting a deficiency in BDNF might contribute to depression-like behaviors (Duman and Monteggia, 2006). Emerging studies have indicated that BDNF plays a critical role in development of depression and antidepressant treatment (Nawa et al., 1994; Shimizu et al., 2003; Brunoni et al., 2008; Hammack et al., 2009; B. H. Lee and Kim, 2010). These studies indicated that compromised hippocampal neurogenesis induced by decreased BDNF signaling might partially contribute to the depression-like behaviors. However, the possibe effects of CS on behaviors and the molecular mechanism underlying the fact that early-life CS exposures affects brain function still remains undefined.

Recent studies from Jamal’s group revealed significant associations of cigarette smoking and BDNF concentrations in serum (Jamal et al., 2015b). Furthermore, they found that BDNF Val(66)Met may moderate depression or anxiety from smoking (Jamal et al., 2015a). Thus, we hypothesized that susceptibility to CS exposure exists during prenatal and early postnatal periods and that CS exposure during these “critical periods” may alter BDNF/TrkB signaling and induce depression-like behaviors in later life. The present experiments are designed to identify critical developmental periods of susceptibility to CS exposure in mice and characterize changes in behaviors and neurotrophins expression during these critical periods to determine the possible underlying mechanisms.

## Methods

All procedures were performed in accordance with the recommendations of the *Guide for the Care and Use of Laboratory Animals 8* ^*th*^ *edition*, published by the National Institutes of Health in 2011, and the protocols were approved by the local Animal Care and Use Committee.

**Figure 1.**
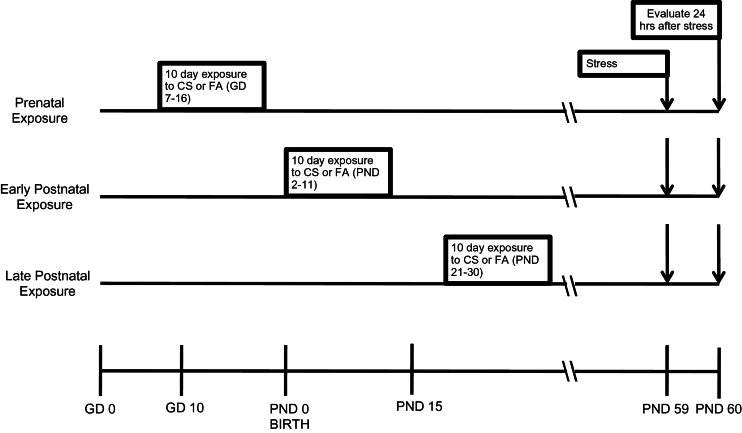
Time line for cigarette smoke (CS) exposure and experimental measurement. Prenatal exposures occurred at gestational day (GD) 7–16; early postnatal exposure occurred at postnatal day (PND) 2–11; and late postnatal exposure occurred PND 21–30. All groups were exposed to acute restraint stress for 1 hour on PND59 and followed by behavioral testing 24 hours later. Brain samples were harvested for all the other evaluations after the last behavioral testing.

The central nervous system is not fully developed during prenatal life, and the structural and functional development continues until postnatal days (PND) 14 to 21 days (Arnold and Trojanowski, 1996; Grove and Tole, 1999; Tole and Grove, 2001). The effects of CS exposure were investigated in 3 different ages of imprinting control region (ICR) mice: gestational day (GD) 7 to GD 16, which corresponds to the period of human fetal development; PND 2 to PND 11, which corresponds to the human neonatal period; and PND 21 to PND 30, which is similar to the human prepubertal time period (Pinkerton and Joad, 2000). The previous studies have shown that CS exposure for 6h/d for a few weeks to 2 months alters pulmonary function, immune responses, and brain neurons in monkey, rat, and mouse models (Barrett et al., 2002; Yu et al., 2008; Wu et al., 2012). Three different age of mice were exposed to either CS or filtered air (FA) for 6h/d for 10 consecutive days beginning on GD7 (by maternal exposure), PND2, or PND21 (direct exposure) (Figure 1). The 10-day exposure periods were chosen to allow testing of our experimental design in nonoverlapping developmental time periods. In addition, mice at 2 weeks of age are nearing maturity; thus, exposure in the PND2 group was completed before maturity, and exposure in the PND21 began after maturity. During the exposure period, dams and infants lived in the same cage with access to food and water. For the GD7 group, exposure initiated 10 days prior to the approximate date of parturition during the period when the central nervous system is beginning to form. After exposure, mice were housed with access to food and water ad libitum in an FDA-approved facility.

The level of cotinine in blood was measured at the end of the daily CS exposure on GD 7, GD 10, and GD 15 in the GD7 group from dams, on PND 3, PND 6, and PND 10 in the PND2 group from pups, and on PND 22, PND 25, and PND 29 in the PND21 group from exposed mice by cotinine Elisa Kit (Sigma, St. Louis, MO).

All groups of mice received acute restraint stress (ARS) for 1 hour on PND59 and then were challenged with behavioral tests 24 hours later. After behavioral tests, mice were sacrificed by decapitation. The hippocampus and prefrontal cortex were dissected and immediately frozen on dry ice and kept at -80°C for PCR array, Western blotting, and ELISA test. The intent of this design was to examine the effects of CS on behaviors and potential mechanisms associated with early-life exposure by comparing responsiveness to CS after a period of recovery in naive mice and mice exposed to CS at 3 stages of life. ARS for 1 hour could not decrease exploration in the open arm of elevated plus-maze (Vargas-Lopez et al., 2015). In our preliminary experiment, we also found that ARS in our stress model for 1 hour did not produce significant endocrinological or behavioral changes in naive mice. Restraint stress for a minimum 4 hours was required to produce depression-like and anxiety-like behaviors. To test whether CS exposure increased the vulnerability and risk of depression-like behaviors, mice were challenged with ARS for 1 hour, and behavioral tests were performed 24 hours later.

### CS Exposure

The CS exposure with the same exposure equipment and methods was used in our studies as described previously (Wu et al., 2009, 2012). Briefly, mice were randomly placed in an exposure chamber (BioClean, DuoFlo, model H 5500, Lab Products Inc) that measured 1.92 x 1.92 x 0.97 m (3.58 m^3^). The mice were housed in separate cages located at the exposure chamber. CS generated from 3R4F Kentucky Reference Research Cigarettes was introduced into the exposure chamber at a rate of 4 cigarettes every 15 minutes for 6h/d using a smoking machine (RM 1/G, Heinr Borgwald GmbH, Hamburg, Germany). The smoking machine can produce isolated side-stream smoke (passive tobacco smoke) or a combination of mainstream and side-stream smoke (active tobacco smoke). For the present study, pregnant mice were exposed by combination of mainstream and side-stream smoke (active tobacco smoke), and PD2 and PD21 groups were exposed by side-stream (passive) smoke. At the end of the 6-hour exposure period, the exhaust fan on the BioClean unit was turned on to rapidly lower the level of CS in the exposure chamber. The mice were then transported to the animal facilities. The concentrations of carbon monoxide in the exposure chamber were monitored and kept at an average of about 50 parts per million (ppm). Relative humidity was about 50% and temperature was about 23°C. Total suspended particulate concentration was about 1.1mg/m^3^. Control animals were sham exposed to FA in whole body inhalation chambers under identical conditions (temperature, humidity and flow rate) to CS exposed mice.

### Behavioral Procedures

Before the behavioral testing, animals were habituated for 1 week by being transferred from the housing room to the testing room, being allowed to sit for 45 minutes, gently handled, and then returned to the housing room. The behavioral tests were conducted in the following order 24 hours after ARS: open field test (OFT), forced-swim test (FST), and tail-suspension test (TST) on 3 consecutive days.

#### ARS

The experiment was conducted as described previously (Hare et al., 2014). Briefly, mice were restrained in 50-mL centrifuge tubes modified to allow air circulation for 1 hour. Mice were not physically squeezed and not able to move forward or backward. Mice were deprived of food and water during the entire period of exposure to stress.

#### OFT

The OFT was performed as previously described (Li et al., 2009; Masood et al., 2009). The open field was made of white acrylic (50×50) with 22-cm-high walls. The floor was divided into 16 squares by black parallel and intersecting lines. Mice were placed individually in one corner of the open-field and entry latency (time to enter the rest of the open field from the start location), ambulation (with all 4 paws placed into a new square), and rearing (with both front paws raised from the floor) were recorded for 5 minutes. The apparatus was thoroughly cleaned using 70% ethanol after each animal.

#### FST

The FST was described previously (Page et al., 1999). Briefly, a mouse was placed individually in a Plexiglas cylinder (45cm high × 20cm diameter) containing water at 22 to 23°C and at a depth of 28cm. Immobility, which was defined as floating in an upright position without additional activity other than necessary for the mouse to keep its head above the water, was recorded for 6 minutes.

#### TST

The tail suspension test was performed as previously described (Steru et al., 1985). Briefly, each mouse was suspended 50cm above the floor using adhesive tape placed approximately 1cm from the tip of its tail. The duration of immobility was recorded during the 6-minute test period. Mice were considered immobile only when they hung passively and completely motionless.

### Gene Expression of Neurotrophins and Receptors

Mice were sacrificed by decapitation after the last behavioral test. The hippocampus and prefrontal cortex were dissected and immediately frozen on dry ice and kept at -80°C. To determine neurotrophins and their receptors, RNA was extracted using TRIzol reagent (Invitrogen), and a portion (2 μg) of total RNA isolated was treated with Turbo DNase (Turbo DNA-free kit; Ambion) and reverse-transcribed into cDNA with the use of the RT^2^ first strand kit (Qiagen Inc, Valencia, CA) following the manufacturer’s instructions. Mouse neurotrophin and receptors RT^2^ Profiler PCR Array kit (Qiagen Inc) was selected for the present study. PCR Array was performed according to the manufacturer’s protocol. Briefly, an experimental cocktail was prepared for each plate made up of the processed cDNA and 2× instrument-specific and ready-to-use array RT^2^ qPCR master mix, containing SYBR Green and a reference dye. A portion (25 μL) of the experimental cocktail was placed into each well of the PCR array plate containing the predispensed gene-specific primer sets, and PCR was performed on the ABI Prism 7500 Sequence Detection System. A 2-step cycling program was used (10 minutes at 95°C to activate the HotStart DNA polymerase, followed by 40 cycles of denaturing for 15 seconds at 95°C and annealing for 1 minute at 60°C). Data collected was entered into online software PCR Array Data Analysis Web Portal provided by the manufacturer for data analysis. Gene expression levels were normalized against the housekeeping genes, including *GUSB* (glucuronidase β), *HPRT1* (hypoxanthine guanine phosphoribosyl transferase 1), *HSP90AB1* (heat-shock protein α class B member 1), *GAPDH*, and *ACTB* (β-actin). Fold changes in gene expression were calculated using the 2^−ΔΔC^_t_ method using the manufacturer’s software.

### Immunoblotting Analyses

The hippocampus and prefrontal cortex were homogenized in ice-cold RIPA lysis buffer (Upstate, Temecula, CA) and centrifuged at 16 000 × *g* for 30 minutes. Supernatant was mixed with an equal volume of Laemmli sample buffer and heated to 100°C for 2 minutes. Variable volumes of sample containing equal amounts of protein were loaded onto gels for SDS-PAGE. Following separation by electrophoresis, proteins in the gels were transferred to nitrocellulose membranes, which were incubated with rabbit phospho-TrkB antibody (1:500; Santa Cruz, Dallas,Tx) or β-actin antibodies (1:1000; Chemicon, Temecula, CA) overnight at 4°C and then with Alexa Fluor 680-conjugated secondary antibody (1:20 000; Invitrogen) for 30 minutes at room temperature. The detection and quantification of specific bands were carried out using a fluorescence scanner (Odyssey Infrared Imaging System, LI-COR Biotechnology, Lincoln, NE).

### Enzyme-Linked Immunosorbent Assay (ELISA)

Hippocampus and prefrontal cortex were homogenized in ice-cold lysis buffer (Upstate,Temecula, CA) and then centrifuged at 16 000 × *g* for 20 minutes (4°C). The supernatants were subsequently analyzed by ELISA for BDNF (Promega, Madison, WI) according to the manufacturer’s instructions. All samples were run in triplicate, and as a negative control, a Phosphate-buffered saline sample was run with each assay.

### Data Analysis

Unless otherwise stated, results are expressed as mean ± SEM. Statistical analyses of behavioral test, ELISA, and Western blotting were performed using 2-way ANOVA followed by Bonferroni’s posthoc test. One factor is age, and another factor is CS exposure. The data of PCR array were calculated using the 2^−ΔΔC^_t_ method using the manufacturer’s software. *P* < .05 was considered significant, and n represents the number of animals studied.

## Results

### Effect of the Prenatal and Postnatal CS Exposure on Depression-Like Behaviors

The level of serum cotinine was monitored at GD 7, GD 10, and GD 15 in GD7 group from dams, PND 3, PND 6, and PND 10 in PND2 group from pups, and PND 22, PND 25, and PND 29 in PND21 group (Table 1).

**Table 1.** The Levels of Cotinine (ng/mL) at Different Times during CS or FA Exposure

	First Monitoring		Second Monitoring		Third Monitoring	
	CS	FA	CS	FA	CS	FA
GD7	46.01±5.18	0.21±0.08	49.99±6.08	0.19±0.09	51.98±5.13	0.20±0.08
PND2	50.98±5.14	0.20±0.09	51.97±6.69	0.22±0.08	52.96±6.53	0.20±0.09
PND21	48.01±6.30	0.19±0.08	49.99±6.08	0.19±0.09	55.44±6.21	0.22±0.09

Data are means ± SEM. n= 5 mice samples/monitoring/group.

**Figure 2.**
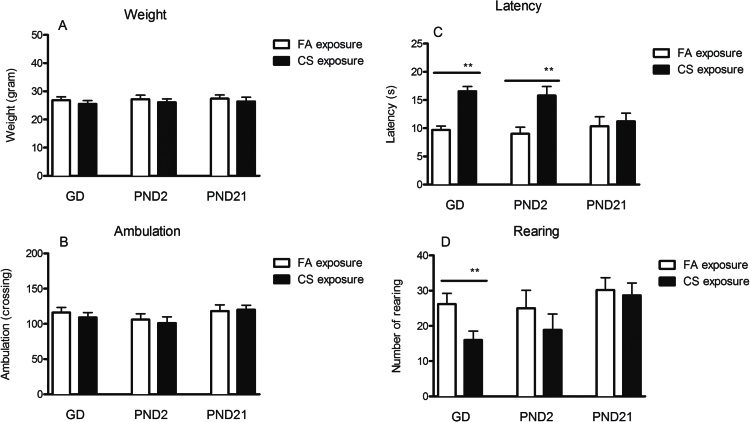
Effects of the prenatal and postnatal cigarette smoke (CS) exposure on weight (A), ambulation (B), latency (C), and rearing (D) in the open field test (OFT). Data shown represent means ± SEM; n = 9/group. *P < .05, **P < .01, Significant difference comparing corresponding data between filtered air (FA) and CS animals.

Before the experiment on PND 59, the weights (mean ± SEM) of CS exposure in GD7 (25.5±1.2g, n = 9), PND2 (26.1±1.1g, n = 9), and PND21 (26.4±1.4g, n = 9) were not significantly different from corresponding FA control groups (GD7: 26.6±1.2g, n = 9, t = 0.779, P = .436; PND2: 27.1±1.4g, n = 9, t = 0.597, *P* = .559 and PND2: 27.2±1.3g, n = 9, t = 0.523, *P* = .608, respectively) (Figure 2A).

In the OFT, there was no significant difference in ambulation between the FA and CS exposure groups at any time period (GD7, PND2, or PND21) (Figure 2B). However, the prenatal and early postnatal (GD7 and PND2), but not PND21 CS exposure, significantly increased entry latency by 70.61% (t = 5.66, *P* < .001) and 75.13% (t = 5.24, *P* < .001), respectively, compared with their FA exposure groups (Figure 1C). Only GD7 CS exposure induced a decrease by 38.93% in rearing (t = 7.36, *P* < .001). In contrast, both PND2 and PND21 CS exposure did not result in any changes in numbers of rearing (Figure 2D).

**Figure 3.**
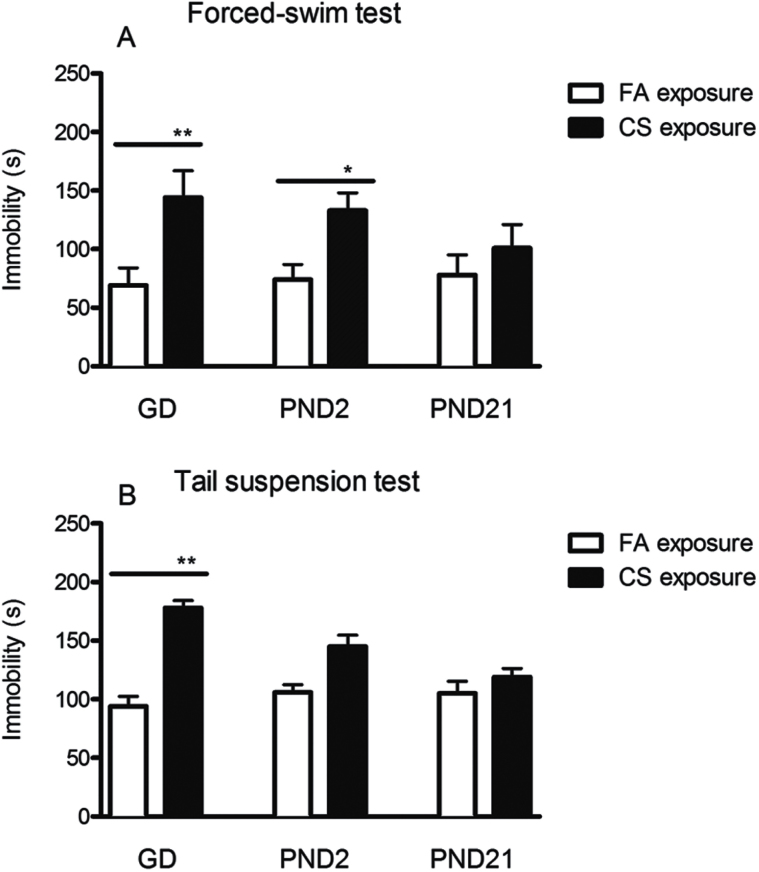
Effects of the prenatal and postnatal cigarette smoke (CS) exposure on immobility in forced-swim test (FST) (A) and tail suspension test (TST) (B). Immobility in gestational day (GD)7 CS exposure groups was increased in both TST and FST, indicating depression-like behaviors. Data shown represent means ± SEM; n = 9/group. *P ≤ 0.05, **P < .01 Significant difference comparing corresponding data between filtered air (FA) and CS groups.

In the FST, immobility of GD7 and PND2 CS exposure groups was significantly increased by 108.69% (t = 7.28, *P* < .001) and 79.72% (t = 4.69, *P* = .024) respectively compared with their FA exposure. By contrast, immobility was not changed significantly by CS exposure in the PND21 groups (Figure 3A). A similar pattern of changes was also found in the TST (Figure 3B). Immobility was significantly increased by 89.36% in the GD7 CS exposure group in comparison with FA control (t = 7.28, *P* < .001). However, PND2 CS exposure only increased immobility by 41.79% compared with FA exposure control, but not significantly (t = 3.01, *P* = .061). In addition, no significant differences were found in PND21 CS exposure groups compared with FA control. These findings suggest that the initial exposure to CS during the prenatal or early postnatal period significantly enhanced depression-like behaviors in offspring.

### Effect of the Prenatal and Postnatal CS Exposure on mRNA Levels of Neurotrophins and Receptors

**Figure 4.**
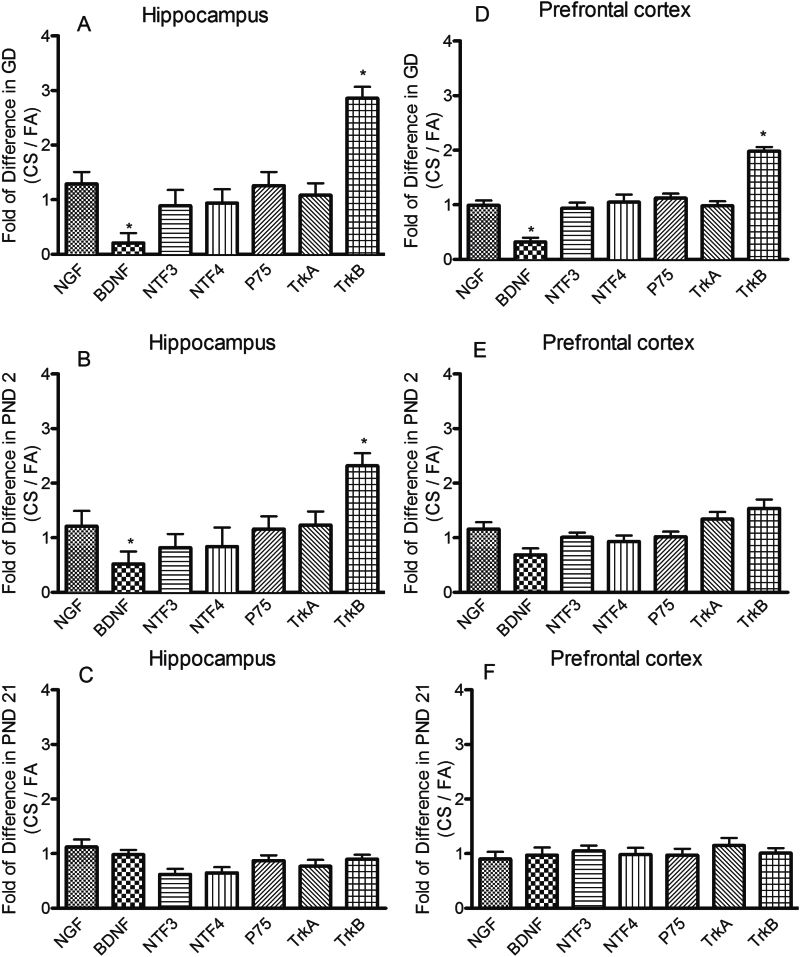
Effects of the prenatal and postnatal cigarette smoke (CS) exposure on gene expression of neurotrophins and their receptors in the hippocampus (A-C) and prefrontal cortex (D-F). Neurotrophins and their receptors including nerve growth factor, brain derived neurotrophic factor (BDNF), neurotrophin-3 (NT-3), neurotrophin-4, low-affinity nerve growth factor receptor P75, neurotrophic tyrosine kinase receptor A (TrkA), and neurotrophic tyrosine kinase receptor B (TrkB) were measured by RT^2^ Profiler PCR Array. Data shown represent means ± SEM; n = 6/group. *P ≤ .05, **P < .01 vs filtered air (FA) exposure.

The gene expressions of neurotrophins and their receptors in the hippocampus and prefrontal cortex were tested using RT^2^ Profiler PCR Array. As shown in Figure 4A and D, CS exposure in the GD7 group significantly decreased BDNF mRNA by 79% (*P* = .021) and 68% (*P* = .024) respectively in the hippocampus and prefrontal cortex compared with their FA controls. In addition, TrkB mRNA was increased by 186% (*P* = .001) and 98% (*P* = .006) in the hippocampus and prefrontal cortex, respectively, in comparison with FA exposure groups. In PND2 group, CS exposure also induced a significant decrease in BDNF mRNA by 48% (*P* = .038) and an increase in TrkB mRNA by 132% (*P* = .003) by compensation in the hippocampus. However, no similar changes in either BDNF mRNA or TrkB mRNA were found in prefrontal cortex (Figure 4B,E). In the PND21 group, CS exposure did not produce any significant changes in mRNA levels of any neurotrophins and their receptors (Figure 4C,F). Other genes analyzed in GD7, PND2, and PND21, which included nerve growth factor, neurotrophin-3, neurotrophin-4, and neurotrophic tyrosine kinase receptor A, did not demonstrate any significant changes in CS exposure groups in comparison with FA control groups.

### Effect of the Prenatal or Postnatal CS Exposure on BDNF Protein Levels

**Figure 5.**
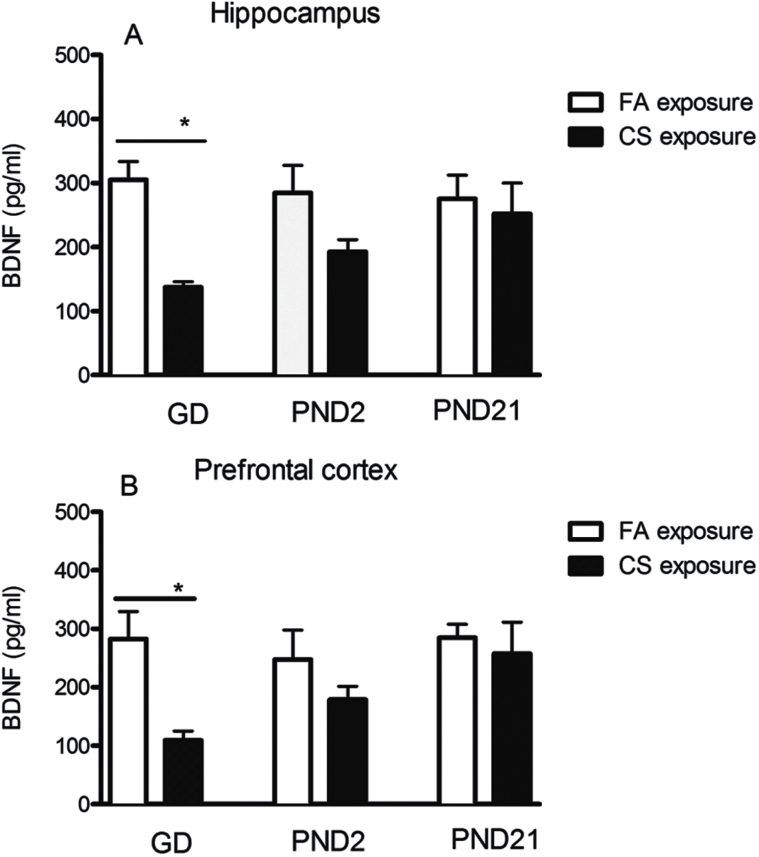
Effects of the prenatal and postnatal cigarette smoke (CS) exposure on protein level of brain derived neurotrophic factor (BDNF) in the hippocampus and prefrontal cortex. Gestation day (GD)7 CS exposure significantly decreased BDNF protein level in both hippocampus (A) and prefrontal cortex (B). Data shown represent means ± SEM; n = 6/group. *P ≤ .05, **P < .01 vs filtered air (FA) exposure.

To measure whether CS exposure influences protein level, BDNF in the hippocampus and prefrontal cortex was measured by ELISA. Data showed that BDNF protein level was significantly decreased in the GD7 CS exposure group by 55.08% (t = 4.52, *P* = .022) (Figure 5A) and 61.26% (t=5.02, *P* = .013) (Figure 5B) in the hippocampus and prefrontal cortex, respectively, compared with FA controls. Similarly, PND2 CS exposure also induced a trend of reduction in BDNF protein level in hippocampus and prefrontal cortex by 32.31% (t = 3.22, *P* = .063) and 27.79% (t = 2.69, *P* = .072) (Figure 5A-B), but not statistically significant. By contrast, PND21 CS exposure did not induce any significant changes in the protein level of BDNF compared with FA control.

### Effect of the Prenatal or Postnatal CS Exposure on Levels of Phosphorylated TrkB

**Figure 6.**
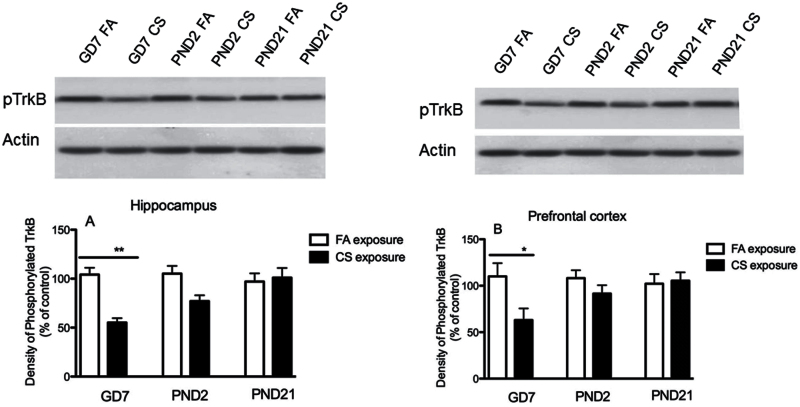
Effects of the prenatal and postnatal cigarette smoke (CS) exposure on phosphorylated neurotrophic tyrosine kinase receptor B (TrkB) in hippocampus (A) and prefrontal cortex (B). Phosphorylated TrkB was significantly decreased in GD7 CS exposure group in comparison with filtered air (FA) control in both hippocampus and prefrontal cortex. Data shown represent means ± SEM; n = 6/group. *P ≤ .05, **P < .01 vs FA exposure.

To determine whether the activation of TrkB receptor is altered as the downstream target of BDNF signaling, phosphorylated TrkB in hippocampus and prefrontal cortex were studied. The results showed that the level of phosphorylated TrkB was significantly decreased by 47.02% (t = 7.69, *P* = .029) in the hippocampus (Figure 6A) and by 42.72% (t = 6.67, *P* = .031) in prefrontal cortex (Figure 6B) in GD7 CS exposure group compared with control. PND2 CS exposure only induced a trend of decrease in phosphorylated TrkB by 26.8% (t = 3.28, *P* = .059) in hippocampus and by 15.3% (t = 2.77, *P* = .074) in prefrontal cortex respectively. In contrast to the GD7 and PND2 groups, PND21 CS exposure did not induce any significant changes in either hippocampus or prefrontal cortex (Figure 6A-B).

## Discussion

Recent studies have shown that maternal smoking during pregnancy and childhood secondhand smoke may increase risk for depression in later life (Herrmann et al., 2008; Cornelius et al., 2011; Mbah et al., 2013; Elmasry et al., 2014; Holz et al., 2014; Yochum et al., 2014). Epidemiological studies also found that children are more susceptible to adverse behavioral effects of passive smoking than adults (Herrmann et al., 2008; Cornelius et al., 2011; Holz et al., 2014), suggesting that exposure to environmental tobacco smoke in early life might be a predisposing factor for such conditions. The results obtained from the current study showed that the changes of behaviors were significantly different between the GD7 CS exposure and control groups, and the levels of BDNF and phosphorylated TrkB in hippocampus were significantly decreased in mice initially exposed to CS during early periods of development (prenatal and early postnatal period). However, initial exposure to CS during a late period of development (PND21) did not appear to affect depression-like behaviors and BDNF/TrkB in hippocampus, suggesting that a critical period of susceptibility exists during prenatal and early postnatal periods.

During the prenatal and early postnatal stage of life, the central nervous system is not fully developed. The sensitivity of the central nervous system in newborns is higher than in adults (Larsen, 1993). This might be due to the level of BDNF in brain during the early development period. BDNF is lowest during the embryo period and then gradually increases into adulthood, especially in the hippocampus (Maisonpierre et al., 1990), which is consistent with the timing of robust neurogenesis in the central nervous system (Altman and Bayer, 1984; Lehuen et al., 1990). Throughout the development of the central nervous system, BDNF is responsible for proliferation and maintenance via regulating neuronal plasticity, influencing neuronal differentiation, and survival (Soule et al., 2006; Cowansage et al., 2010). Mice lacking BDNF demonstrated severe deficiencies in central nervous system development (Ernfors et al., 1994; Pozzo-Miller et al., 1999). Thus, dysregulated BDNF level by CS exposure during the critical period of rapid brain growth may alter the normal developmental process and lead to long-term cellular alterations that result in the inability of the brain to make appropriate adaptive responses (Cornelius and Day, 2009) and contribute to the depression-like behaviors in later life.

Previous studies have reported that mice with deficiency in BDNF demonstrated depression-like behaviors, and administration of BDNF produced antidepressant effects (Nawa et al., 1994; Karege et al., 2002; Shimizu et al., 2003; Karege et al., 2005; Brunoni et al., 2008; Hammack et al., 2009; B. H. Lee and Kim, 2010), indicating BDNF correlates with development of depression. In addition, plasma level of BDNF in patients with major depression was significantly lower than healthy participants (Gonul et al., 2005; Karege et al., 2005; Brunoni et al., 2008), and chronic antidepressant treatment significantly relieved the clinical symptoms by increasing BDNF (Shimizu et al., 2003) and activation of TrkB in the brain (Castren et al., 2007; Monteggia et al., 2007; B. H. Lee and Kim, 2010). These findings are consistent with our finding that prenatal and early postnatal (GD7 and PND2) CS exposure decreased BDNF level and TrkB activation and increased depression-like behaviors. However, other studies have shown that deficiency of BDNF or TrkB cannot induce depressed behaviors, although BDNF is required for effective antidepressant treatment and recovery of neuronal networks (Nawa et al., 1994; Pozzo-Miller et al., 1999; Saarelainen et al., 2003; B. H. Lee and Kim, 2010; Papaleo et al., 2011). Therefore, further studies are needed to investigate whether BDNF or TrkB receptor agonist can reverse abnormal neuronal networks and behavioral changes induced by CS exposure. Recent studies found that glial cell derived neurotrophic factor promoted development of neurons and played an important role in the pathogenesis of mood disorders (Barbosa et al., 2011; Tunca et al., 2014, 2015). It is not clear whether it functions simultaneously as BDNF via same mechanism in CS exposure-induced abnormalities during early life and needs further investigation in the future.

Structural and morphological abnormalities in specific brain regions such as prefrontal cortex and hippocampus are associated with depression. MRI studies showed that depressed patients had significantly decreased left and right hippocampal volumes compared with controls (Harrison, 2002; Campbell et al., 2004). PET imaging studies also demonstrated that prefrontal lobe hypometabolism in primary and secondary depression correlated with the degree of frontal inactivity in clinical patients (Kimbrell et al., 1999; Hastings et al., 2004). In animals, the reduction in volume might be due to abnormal cellular developments, including decreased neurogenesis, loss of glial cells, and retraction of dendrites. In addition, abnormal neural circuits and structure (Folstein et al., 1985; Phillips et al., 2003) and imbalance of activity in prefrontal cortex and hippocampus contribute to the depression (Koenigs and Grafman, 2009). These studies from both clinical and animal models have demonstrated that hippocampus and prefrontal cortex play an important role in depression. Our results showed that prenatal CS exposure decreased BDNF/pTrkB in both hippocampus and prefrontal cortex. Especially, GD7 and PD2 CS exposure downregulated BDNF/TrkB in hippocampus, while only GD7 CS exposure decreased BDNF/TrkB in prefrontal cortex significantly, suggesting hippocampus and prefrontal cortex are more sensitive to CS exposure during the prenatal period and may produce prolonged effects on brain morphology or neuronal plasticity due to deficiency of BDNF. These findings parallel clinical studies that CS exposure reduced cortical gray matter in teenagers (Rivkin et al., 2008) and decreased frontal cortex and hippocampal gyrus in adolescents (Toro et al., 2008; Lotfipour et al., 2009), indicating the effect of CS exposure on the brain might be in a region-specific manner or a time-specific manner throughout the neural development (El Marroun et al., 2014).

Nicotine and carbon monoxide are of major concern because of their known toxic actions and their responsibility for most of the harmful effects during the perinatal CS exposure. Cotinine is the major proximate metabolite of nicotine and has been widely used as a biomarker in active and secondhand tobacco smoke. The average level of cotinine in our experiment was around 50ng/mL, which was similar with the cotinine level typically found in human light smokers (30–100ng/mL) and less than the cotinine level in 17-cigarette/d smokers (average 122ng/mL) (Pirkle et al., 1996; Benowitz et al., 2009). Serum cotinine level reflects nicotine level after recent exposure to CS. Nicotine can cross biological membranes including the placental barrier into the fetal and blood brain barrier. Navarro et al. (1989) showed that prenatal exposure to high doses of nicotine via maternal infusions impaired nervous system development. Eriksson et al. (2000) also found that exposure to nicotine in the prenatal period stimulated the nicotinic acetylcholine receptor in brain and modified behavior of mice later in life. In rat models, exposure to nicotine during utero has demonstrated behavioral, neurochemical, and cognitive abnormalities in offspring (Bertolini et al., 1982; Levin et al., 1996). Neonatal exposure to nicotine also elicited neurobehavioral defects in adult (Ankarberg et al., 2001). These studies indicate that nicotine potentially alters normal brain growth or nicotinic acetylcholine receptor during the prenatal and early postnatal period, which may subsequently affect behaviors later in life. Carbon monoxide can also cross the placenta and combines reversibly with hemoglobin to form carboxyhemoglobin in both maternal and fetal blood. It is also well known that the reduction of oxygen and the increase of carbon monoxide during CS exposure are detrimental to brain development (Caravati et al., 1988; Fogh-Andersen et al., 1988; Parslow et al., 2004; J. H. Lee et al., 2012). Due to the complexity of CS exposure, we could not identify specific components involved in the abnormalities in our study.

Previous studies have found that significant strain differences exist for variables used to measure depression-like behaviors in mice models such as FST and TST (Lucki et al., 2001; Mineur et al., 2006; Sade et al., 2014). C57BL/6 mice demonstrate a decrease in immobility in TST, whereas BALB/C mice show an increase in immobility after chronic mild stress (Mineur et al., 2006). A study comparing 11 mouse strains in the FST showed that there is a 10-fold range of immobility values (Lucki et al., 2001). These suggest the existence of substantial behavioral differences between mouse strains in the baseline performance. ICR mouse is a well-established animal model that has been used extensively for studying behavioral profiles (Sade et al., 2014; Avitsur et al., 2015; Yokota et al., 2015). Recent studies showed that prenatal exposure to diesel exhaust particles and fluoxetine affects behavioral response in ICR mice (Avitsur et al., 2015; Yokota et al., 2015). Thus, ICR mice were used in our behavioral studies. In addition, gender differences exist in the etiology and responses to stress in mice (Palanza, 2001; Frye and Walf, 2009). Emerging evidence has demonstrated that antidepressant-induced behavioral effects vary with the estrous cycle in female animals (Carrier and Kabbaj, 2013; Franceschelli et al., 2015), indicating that gonadal hormones can at least partially contribute to the differences of responses to antidepressant treatment between male and female rodents. To avoid the influence of gonadal hormones to our experiment, male ICR were used in our study.

In conclusion, the results showed that exposure to CS during prenatal (maternal exposure) and early postnatal life increased the depression-like behaviors and decreased BDNF/TrkB signaling later in life. Interestingly, these responses were not observed when CS exposure occurred in late postnatal life (near puberty in mice), suggesting that a critical period of susceptibility to CS exposure exists in the prenatal and early postnatal period of brain development in mice, which results in a downregulation in BDNF/TrkB signaling in the hippocampus and enhances depression-like behaviors later in life.

## Statement of Interest

None.
